# Homoepitaxial Growth of Metal Halide Crystals Investigated by Reflection High-Energy Electron Diffraction

**DOI:** 10.1038/srep40542

**Published:** 2017-01-10

**Authors:** Pei Chen, Padmanaban S. Kuttipillai, Lili Wang, Richard R. Lunt

**Affiliations:** 1 Department of Chemical Engineering and Materials Science, Michigan State University, East Lansing, MI, 48824, USA; 2 Department of Physics and Astronomy, Michigan State University, East Lansing, MI, 48824, USA

## Abstract

We report the homoepitaxial growth of a metal halide on single crystals investigated with *in situ* reflection high-energy electron diffraction (RHEED) and *ex situ* atomic force microscopy (AFM). Epitaxial growth of NaCl on NaCl (001) is explored as a function of temperature and growth rate which provides the first detailed report of RHEED oscillations for metal halide growth. Layer-by-layer growth is observed at room temperature accompanied by clear RHEED oscillations while the growth mode transitions to an island (3D) mode at low temperature. At higher temperatures (>100 °C), RHEED oscillations and AFM data indicate a transition to a step-flow growth mode. To show the importance of such metal halide growth, green organic light-emitting diodes (OLEDs) are demonstrated using a doped NaCl film with a phosphorescent emitter as the emissive layer. This study demonstrates the ability to perform *in situ* and non-destructive RHEED monitoring even on insulating substrates and could enable doped single crystals and crystalline substrates for a range of optoelectronic applications.

Alkali halide crystals are widely used in many optical and optoelectronic applications. NaCl crystals are used as one of the most common infrared transmission windows for spectrophotometers and useful for energy detection in x-ray monochromators^1^,^2^. Doped NaCl bulk crystals have been used for single crystal emitters and scintillation detectors^3^,^4^,^5^ and thin film NaCl can be used as buffer layers for organic light-emitting devices (OLEDs)^6^,^7^. NaCl crystal surfaces have been used for studying epitaxial growth of organic and inorganic thin films^8^,^9^,^10^,^11^,^12^. The large range of lattice constants of alkali halide crystals could also enable growth of epitaxial metal-halide perovskites. Thus, understanding fundamental aspects of homoepitaxial metal halide growth could have a significant impact.

Among the techniques for studying epitaxial growth, reflection high-energy electron diffraction (RHEED) is extremely powerful for *in situ* and real-time monitoring. Due to the grazing incidence (1–3°) of the electron beam at the sample surface, the penetration depth (5–10 Å) is at the monolayer scale, making it an extremely surface sensitive technique. RHEED intensity oscillations have been routinely used during epitaxial growth of metals and semiconductors to monitor the growth rate^13^,^14^, understand crystal growth mechanisms and surface reconstructions^15^,^16^,^17^,^18^, estimate surface diffusion^19^,^20^,^21^, and measure dopant incorporation^22^,^23^. A better understanding of the characteristics of the oscillations would provide insight into the microscopic processes during epitaxial growth. Although significant progress has been made in studying RHEED oscillations in epitaxial growth on metals^24^,^25^,^26^,^27^ and semiconductors^28^,^29^,^30^,^31^, there are no reports on halide insulators. Hetero- and homoepitaxial growths of alkali halides using molecular beam epitaxy (MBE) have been studied by both electron and He atom scattering, however with a limited growth parameter range in ultrahigh vacuum^32^,^33^,^34^,^35^,^36^,^37^,^38^. In the case of the studies with RHEED in MBE, electron beams had to be removed within seconds to avoid charging, therefore limiting the ability to monitor oscillations and to obtain real time growth mode information. In the case of the studies with He scattering, very little variation was found as a function of growth conditions. In this work, NaCl homoepitaxial growth on NaCl (001) surfaces is performed by thermal evaporation and studied using both *in situ* ultra-low current RHEED and *ex situ* atomic force microscopy (AFM), which reveals strong variations in the growth modes as a function of growth conditions. The deposition and monitoring technique demonstrated here for studying alkali halides could have implications for the epitaxial growth of halide perovskites which has tremendous potential for future electronic devices^39^,^40^,^41^,^42^,^43^,^44^.

## Experimental

Vapor deposition of NaCl was performed in a multisource thermal evaporator (Angstrom Engineering) equipped with real-time and *in situ* RHEED system (STAIB Instruments) operated with an ultra-low (<10 nA) current and beam energy of 30.0 keV. By using ultra-low current, the damage and charging of the film is negligible over the growth times investigated enabling the use of this technique on organic and insulating surfaces^45^. RHEED oscillations are monitored with substrates fixed at various in-plane orientations (KSA400). Rotation dependent patterns were collected after growths were halted. The NaCl single crystal substrates were freshly cleaved in a glovebox prior to deposition with the (001) surface exposed and attached to the substrate holder using conductive tape. Epitaxial growth was performed under a base pressure less than 3 × 10^−6^ torr and deposition rates were measured *in situ* with a quartz crystal microbalance and calibrated *ex situ* by cross-sectional scanning electron microscopy (SEM) using single crystal potassium bromide as a substrate for layer contrast. The heating/cooling stage inside the chamber was used to pre-heat/cool the NaCl single crystal substrates to the required temperature before deposition. Substrate temperature was monitored and controlled (Watlow EZ-Zone) with a thermocouple integrated into the substrate heating/cooling plate that was calibrated to the surface temperature using an *in situ* non-contact infrared-to-analog converter module (Omega OSM101). AFM was performed in contact mode for *ex situ* film morphology characterization. A silicon tip coated in Ti/Ir was used for the AFM measurements (Asylum Research). Organic light-emitting diodes (OLEDs) utilizing NaCl as the host were fabricated on degreased substrates coated with indium tin oxide(ITO) using the following structure: ITO/MoO_3_ (150 Å)/NaCl:Ir(ppy)_3_ (6 vol.%, 200 Å)/LiQ(8 Å)/Al (800 Å) where Ir(ppy)_3_ is *fac*-tris(2-phenylpyridine)iridium and LiQ is 8-hydroxyquinoline lithium. The control device used the same structure without any emitter in the NaCl. The reference conventional device structure is: ITO/α-NPD (400 Å)/CBP: Ir(ppy)_3_ (6 vol.%, 200 Å)/BCP (75 Å)/Alq_3_ (200 Å)/LiF(8 Å)/Al (800 Å), where α-NPD is 4,4′-bis[N-(1-naphthyl)-N-phenyl-amino]biphenyl, CBP is 4,4′-N,N′-dicarbazole-biphenyl and Alq_3_ is tris-(8-hydroxyquinolinato) aluminum. Electrical characterization was performed using a digital source meter (Keithley 2420) and a picoammeter (Keithley 6487). Luminescence current was measured using a large area Si photodetector (Hamamatsu) and electroluminescence (EL) spectra were measured using a calibrated spectrometer (Ocean Optics USB 4000).

## Results

**Figure 1:**
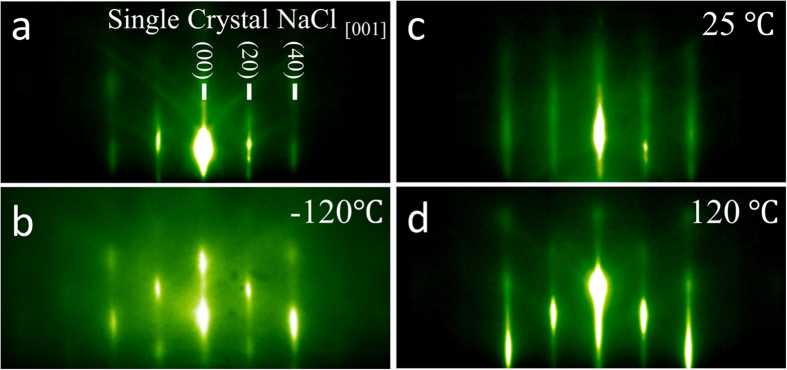
Temperature dependent *in situ* reflection high energy electron diffraction (RHEED) of NaCl homoepitaxy. Diffraction patterns for (**a**) bare single crystal NaCl cleaved along the (001) plane; homoepitaxial growth of 200 Å NaCl at (**b**) −120 °C, (**c**) 25 °C and (**d**) 120 °C. Note that the Kikuchi lines and continuous streaky patterns of the NaCl persist after growth at 25 °C and 120 °C, while at −120 °C Kikuchi lines disappear and streaky pattern becomes discontinuous after growth.

**Figure 2:**
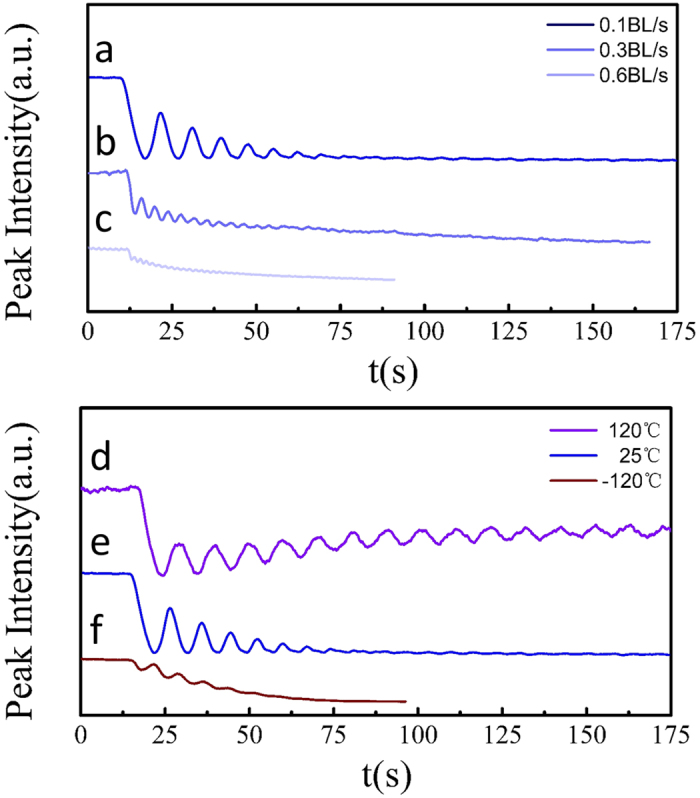
NaCl homoepitaxial growth mode RHEED oscillations. Room temperature growths as a function of growth rate: (**a**) 0.1 (±0.02)BL/s, (**b**) 0.3 (±0.05)BL/s and (**c**) 0.6 (±0.1)BL/s; fixed rate growths of 0.1 (±0.02)BL/s as a function of growth temperature (**d**) −120 °C, (**e**) 25 °C and (**f**) 120 °C. RHEED oscillations intensities were recorded from the specular spot and correspond to one unit cell (or BL) per oscillation. Note that (**a**) and (**e**) are the same data.

**Figure 3:**
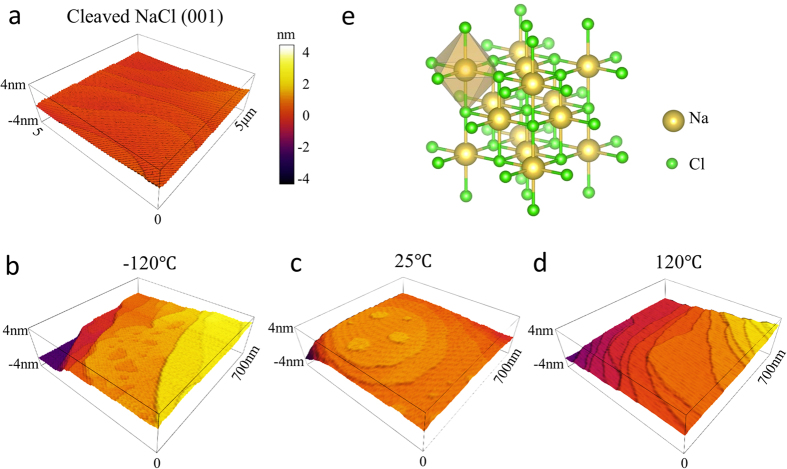
Temperature dependent growth mode and 3D morphology. Atomic force microscopy (AFM) images for (**a**) bare single crystal NaCl and as a function of growth temperature for homoepitaxial growth of 200 Å NaCl at (**b**) −120 °C, (**c**) 25 °C, (**d**) 120 °C. (**e**) Crystal structure of NaCl. Line scans showing step height are included in the Supplementary Information. Two average step heights are observed: 2.5 Å (corresponding to the ML) and 5.0 Å (corresponding to the BL), with the ML steps observed most frequently.

Experiments were performed to study homoepitaxial growth of NaCl on NaCl (001) surfaces as a function of growth rate and temperature. For NaCl, the monolayer (ML) and bilayer (BL) thicknesses are defined as *a*/2 (2.82 Å) and *a* (5.64 Å), respectively, consistent with other BCC and FCC crystals^46^. In the first set, growths were performed at room temperature (25 °C) with different rates to confirm RHEED oscillation interpretation: 0.1 BL/s, 0.3BL/s and 0.6BL/s. In the second set, low (−120 °C) and high temperature (120 °C) growths were performed at a growth rate of 0.1 BL/s as comparison to the room temperature growth to study the effect of temperature on the growth mode. RHEED patterns of NaCl homoepitaxial growth of 200 Å at different temperatures are compared in Fig. 1. A typical RHEED pattern of the NaCl single crystal is shown in Fig. 1(a). The electron beam is directed onto the surface along the NaCl [001] direction. At growth temperatures of 25 °C and 120 °C, the sharp and continuous streak features of the patterns are still present after the growth with a clear single-crystal rotation dependence and Kikuchi patterns still observable. For growth at −120 °C, broken streak patterns are observed with a corresponding disappearance of Kikuchi patterns. Specular RHEED intensities are shown in Fig. 2 for each growth. Depositions performed at 25 °C at each rate all show clear RHEED oscillations with a sudden specular intensity drop when the growth is initiated. The period of the RHEED oscillations is consistent with the deposition rate within the error of the deposition rate recorded by the quartz crystal microbalance (±0.1 Å/s), where each period corresponds to a complete unit cell (or BL) similar to the oscillations observed for stoichiometric FeAl on AlAs^47^. As the growth proceeds, there is an overall damping of the specular intensity shown in Fig. 2(a–f). At a growth temperature of 120 °C, continuous RHEED oscillation are observed for >25 BLs, along with a recovery in the specular intensity during longer deposition time as shown in Fig. 2(d). In contrast, the oscillations observed at low temperature growth at −120 °C persist for only a few BLs as shown in Fig. 2(f). Figure 3 shows the contact mode AFM morphology of 200 Å of homoepitaxial NaCl growth for each growth temperature. The average step heights in these AFM images correspond to either ML (~2.8 Å) or BL (~5.6 Å) steps where ML steps are most commonly observed (see Supplementary Fig. S1). At room temperature, intra-terrace nucleation is clearly evident by the emergence of isolated ML-thick circular islands speckled within terraces. When the growth temperature is reduced to −120 °C, rougher terraces with greater terrace heights (closer to a BL) are found to form during growth along with a large density of ‘voids’. At 120 °C, smooth terraces are formed with smaller terrace width and no observable intra-terrace nucleation.

## Discussion

At room temperature, layer-by-layer growth of NaCl is observed for both low and high rate deposition indicated by strong intensity oscillations. From the rate-dependence, it is clear that highly quality epitaxial growth can be maintained even for the highest rates measured here (0.6 BL/s), which allows the growth of 1 micron thick film in 50 min.

In general, RHEED oscillations stem from interference of the periodic formation and coalescence of 2D nuclei during the deposition of each layer^48^. This mechanism is briefly described: when the deposition is initiated there is a formation of 2D nuclei that creates a disruption in the crystal potential and causes part of the diffraction beam to become out-of-phase. The coalescence of multiple islands into a smooth surface returns the layer to a uniform potential and restores coherence and specular intensity^49^. In the case of NaCl with FCC symmetry, the full period of the RHEED oscillation corresponds to a full unit cell (or BL). This means that the bottom of the oscillation corresponds to a completed half unit cell (or ML) and each ML forms a complete layer prior to the next. This picture is consistent with the top-layer interference model described above where a completed half unit cell has greater destructive interference analogous to the phase mechanism that forbids the (001) and allows (002) diffraction peaks. This also explains the observation of half unit cell (or ML) step heights in AFM images. In the case of perfect layer-by-layer growth on singular surfaces, each layer is completed before nuclei are formed on the next layer – the resulting oscillations would then persist ideally with the same amplitude (undamped) indefinitely. However, in many cases adsorbates begin to nucleate on the previous layer before it is fully completed, resulting in either an accumulating degree of roughness or a lack of oscillation coherence that dampens the overall intensity of the specular spot. For the latter, this specular decay is also typically seen for layer-by-layer growth on vicinal surfaces such as with GaAs homoepitaxy where growth on a singular surface (miscut <1 mrad) results in a nearly constant oscillation amplitude while vicinal surfaces (miscut ~7 mrad) results in an intensity decay^21^. Given the vicinal nature of the cleaved NaCl (001) shown in Fig. 3(a), this is the most likely behavior at 25 °C.

When the growth temperature is increased to 120 °C, the RHEED oscillations persist (with a smaller amplitude) for over 25 BLs and there is an overall recovery of the specular intensity (instead of a decay). This specular recovery is a hallmark of step-flow driven growth^50^. That is, as the temperature and the surface diffusivities of adsorbates increase, eventually only growth from the step edge is favorable and intra-terrace nucleation is suppressed. When the diffusion length of adsorbates becomes large enough to allow adsorbates move freely between the boundaries of the terraces, a roughly constant step density is maintained that flows uniformly across the substrate and results in a RHEED intensity that recovers to a nearly constant value. This growth mode and specular recovery is consistent with the morphology in Fig. 3(d) where only terraces are observed (no intra-terrace islands). The continued observation of weak oscillations suggests that we are close to the transition between layer-by-layer and step-flow growth at 120 °C. Indeed, it is likely that we are observing a mixed-mode growth where growth is initiated at step edges but grows radially across the terrace from these nucleation points. This can be clearly seen in the AFM as apparent triple junctions at the end of steps as well as greater step curvature. Thus, it would be expected to see a superposition of both weak oscillations and an overall intensity recovery.

For the low temperature growth at −120 °C, the Kikuchi features of the base NaCl substrate disappear within a coverage of 200 Å, which suggests the formation of an increasing number of grain boundaries and dislocation density. In this case, the fast drop of oscillation intensity within the growth of less than 5 BLs indicates a transition from layer-by-layer to island (3D) growth. AFM image in Fig. 3(b) shows non-uniform coverage at the terraces, larger terrace step height, and a roughing surface morphology. Interestingly, it was predicted that for alkaline halide epitaxy, diffusivities would be large enough to see RHEED oscillations at growth temperatures above 0.1·T_M_ (absolute melting temperature)^35^, which translates to −166 °C and which is surprisingly consistent with our observations (weak but still present oscillations at −120 °C). This would then place the diffusivity in the 10^−7^–10^−8^ cm^2^/s range at low temperature (−100 °C to −150 °C) and 10^−4^ to 10^−5^ cm^2^/s at room temperature^35^.

**Figure 4:**
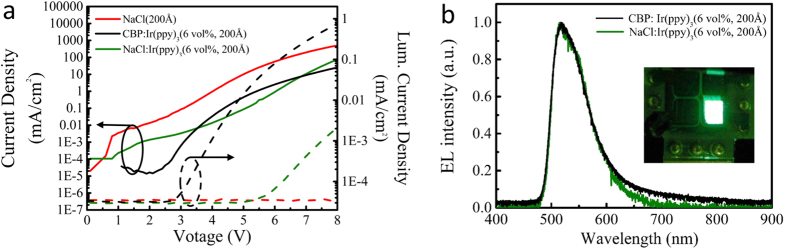
NaCl based OLEDs. (**a**) Current density (*J*) and luminescence current density (*L*) vs. voltage (*V*) of the NaCl, NaCl:Ir(ppy)_3_ and CBP:Ir(ppy)_3_ devices. (**b**) Electroluminescence (EL) spectrum of the conventional CBP:Ir(ppy)_3_ and NaCl:Ir(ppy)_3_ devices. (Inset) Photograph of the NaCl:Ir(ppy)_3_ device showing green electroluminescence.

As a proof of principle demonstration of the potential of NaCl in light emitting electronic devices, we show the incorporation of vapor deposited NaCl as the host in a green phosphorescent OLED structure. Figure 4(a) shows a comparison of current density (*J*) and luminescence current density (*L*) vs. voltage (*V*) characteristics of a control device with undoped NaCl, a device with Ir(ppy)_3_ doped NaCl, and another control device with Ir(ppy)_3_ doped CBP, a well-known host material. The current density curves represent the electrical characteristics while the luminescence current density curves show the optical characteristics of the OLEDs. Figure 4(b) shows the electroluminescence (EL) spectrum of the device, which is consistent with the EL for the conventional host architecture^51^. The absence of luminescence current in the control devices and the matching of the spectrum between NaCl:Ir(ppy)_3_ and CBP:Ir(ppy)_3_ demonstrates that the emission in the doped NaCl devices originates only from the dopant. The optical turn on voltage from the *J-V-L* data for the NaCl doped devices is 5.2 V, which is higher than the expected turn on voltage of 2.4 V for standard CBP host devices. This large optical turn on in the NaCl devices results in lower power efficiency and is likely due to either a higher electron/hole injection barrier or higher series resistance in the unoptimized NaCl device. However, by comparing the current density of doped and undoped NaCl devices, it seems less likely to be related to the series resistance directly and more related to injection barriers since the undoped NaCl exhibits an even higher current density. While the peak external quantum efficiency (EQE), the current-to-forward-emitted-light efficiency, for the NaCl:Ir(ppy)_3_ device is low (0.005%) compared with the conventional host architecture (10%)^51^, the performance could be improved substantially with additional thickness optimization, layer optimization (e.g. exciton blocking layers), layer crystallinity and ordering, variation in the energy levels of the NaCl:emitter pairing, or host co-doping. Additional investigation is necessary to optimize the performance and ultimately understand the connections between resistance, morphology, injection barriers (through variation in dopant energy levels), and emitter-host interactions with this new host system. Considering NaCl can be doped to become conductive (at least at high temperatures)^52^ or epitaxially coupled with highly conductive halide perovskites, these demonstrations suggest new potential pathways to integrate NaCl as a key structural component in epitaxial single crystal optoelectronics.

## Conclusion

We have demonstrated *in situ* and real time RHEED monitoring of homoepitaxial growth of NaCl on NaCl (001). RHEED oscillations and AFM morphologies are observed and discussed to show the impact of growth rate and temperature on changes in growth mode which were previously unrecognized. This study demonstrates the capability of performing RHEED monitoring of epitaxial growth on insulating halide crystals in thermal evaporators. This understanding could be used to enable the mixed growth of alkali halides with tunable lattice constants and enable epitaxial halide perovskite growth for photovoltaic and optoelectronic applications. We have also demonstrated NaCl as a potentially interesting component for OLEDs, further suggesting new opportunities for metal-halide and metal-halide-perovskite based single-crystal epitaxial optoelectronics.

## Additional Information

**How to cite this article**: Chen, P. *et al*. Homoepitaxial Growth of Metal Halide Crystals Investigated by Reflection High-Energy Electron Diffraction. *Sci. Rep.* **7**, 40542; doi: 10.1038/srep40542 (2017).

**Publisher's note:** Springer Nature remains neutral with regard to jurisdictional claims in published maps and institutional affiliations.

## Supplementary Material

Supplementary Information
