# Supplementary material for: Homoepitaxial Growth of Metal Halide Crystals Investigated by Reflection High-Energy Electron Diffraction
Source: Sci Rep. 2017 Jan 10;7:40542. doi: 10.1038/srep40542 (PMC5223175; doi:10.1038/srep40542)
Supplement: Supplementary Information [file srep40542-s1.pdf]

Homoepitaxial Growth of Metal Halide Crystals Investigated by Reflection High-Energy Electron Diffraction

Pei Chen,[1] Padmanaban S. Kuttipillai,[1] Lili Wang,[1] and Richard R. Lunt[1,2,*]

[1] Department of Chemical Engineering and Materials Science, Michigan State University, East Lansing, MI, 48824 USA

[2] Department of Physics and Astronomy, Michigan State University. East Lansing, MI, 48824 USA

*rlunt@msu.edu

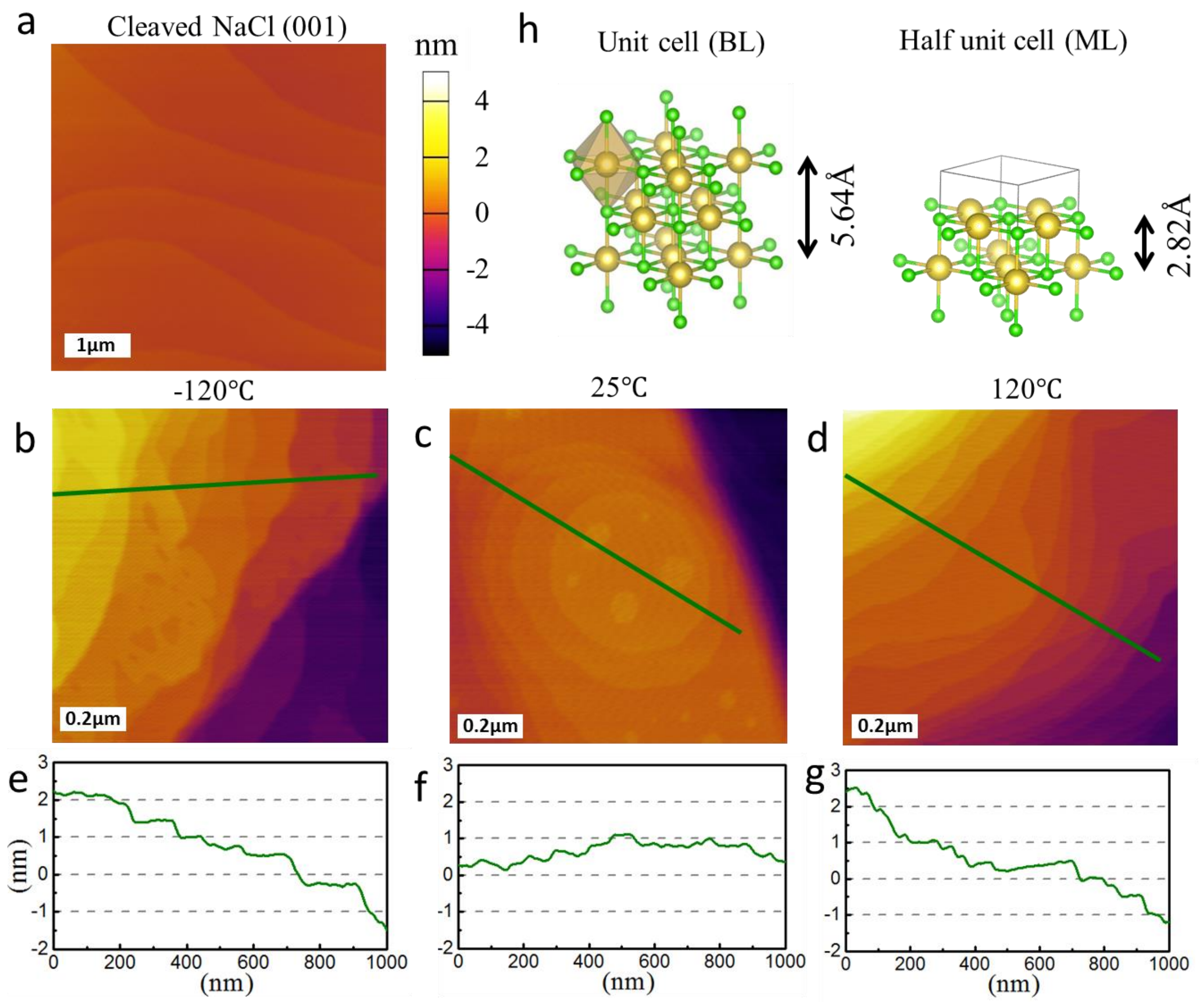

Figure S1. **Temperature dependent growth mode and 2D morphology**. Atomic force microscopy (AFM) images for (a) bare single crystal NaCl and as a function of growth temperature for homoepitaxial growth of 200Å NaCl at (b) -120℃, (c) 25℃, (d) 120℃. (e-g) Corresponding line scans showing step height. (h) Crystal structures for one NaCl unit cell (BL) and half unit cell (ML).

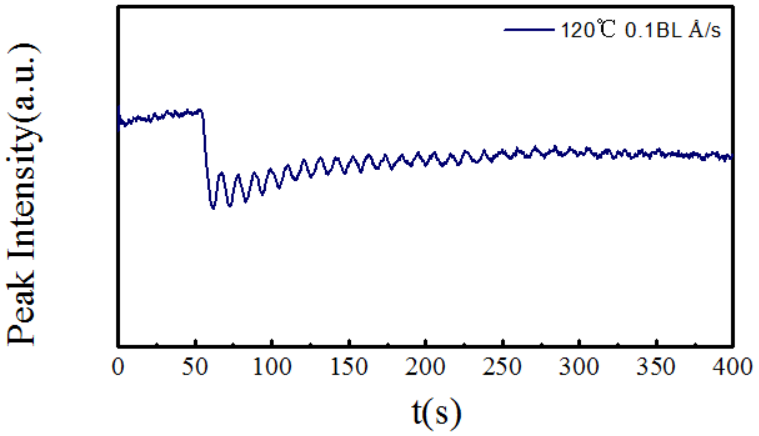

Figure S2. **RHEED oscillation data for NaCl homoepitaxial growth at high temperature.** Complete oscillation data beyond 175s is shown for growth at 120°C, indicating the loss of oscillations after 300s.
